# Principal component and clustering analysis on molecular dynamics data of the ribosomal L11·23S subdomain

**DOI:** 10.1007/s00894-012-1563-4

**Published:** 2012-09-08

**Authors:** Antje Wolf, Karl N. Kirschner

**Affiliations:** 1 Fraunhofer-Institute for Algorithms and Scientific Computing (SCAI), Department Bioinformatics, Schloss Birlinghoven, 53754 Sankt Augustin, Germany; 2 Department of Life Science Informatics, Bonn-Aachen International Center for IT, Rheinische Friedrich-Wilhelms-Universität, Dahlmannstr. 2, 53113 Bonn, Germany; 3 Department of Simulation Engineering, Fraunhofer-Institute for Algorithms and Scientific Computing (SCAI), Schloss Birlinghoven, 53754 Sankt Augustin, Germany

**Keywords:** Clustering, Molecular dynamics, Principal component analysis, Ribosome

## Abstract

With improvements in computer speed and algorithm efficiency, MD simulations are sampling larger amounts of molecular and biomolecular conformations. Being able to qualitatively and quantitatively sift these conformations into meaningful groups is a difficult and important task, especially when considering the structure-activity paradigm. Here we present a study that combines two popular techniques, principal component (PC) analysis and clustering, for revealing major conformational changes that occur in molecular dynamics (MD) simulations. Specifically, we explored how clustering different PC subspaces effects the resulting clusters versus clustering the complete trajectory data. As a case example, we used the trajectory data from an explicitly solvated simulation of a bacteria’s L11·23S ribosomal subdomain, which is a target of thiopeptide antibiotics. Clustering was performed, using K-means and average-linkage algorithms, on data involving the first two to the first five PC subspace dimensions. For the average-linkage algorithm we found that data-point membership, cluster shape, and cluster size depended on the selected PC subspace data. In contrast, K-means provided very consistent results regardless of the selected subspace. Since we present results on a single model system, generalization concerning the clustering of different PC subspaces of other molecular systems is currently premature. However, our hope is that this study illustrates a) the complexities in selecting the appropriate clustering algorithm, b) the complexities in interpreting and validating their results, and c) by combining PC analysis with subsequent clustering valuable dynamic and conformational information can be obtained.

**Electronic supplementary material:**

The online version of this article (doi:10.1007/s00894-012-1563-4) contains supplementary material, which is available to authorized users.

## Introduction

The GTPase-associated region (GAR) on the 70S bacterial ribosome plays a central role in peptide elongation by providing a binding site for elongation factors and by coordinating GTP hydrolysis during protein synthesis [1–3]. The GAR is primarily formed by the ribosomal protein L11 and the helix 43-44 (H43/H44) substructure of the 23S ribosomal RNA. This L11·23S subdomain also binds thiopeptide natural products (e.g., thiostrepton) that have antibacterial activity [4–9], making it a target for rational drug design [10, 11].

L11 is a two-domain protein whose C-terminal domain (CTD) tightly binds the H43/H44 substructure of the 23S rRNA [12]. Its N-terminal domain (NTD) shows a high degree of flexibility [13–16] relative to the CTD and 23S; its differing conformational states may be critical for interaction with translation factors during protein synthesis [3, 6, 17]. Furthermore, the resistance data for thiopeptide antibiotics cannot easily be aligned with a static lock-and-key model for inhibitor binding [18].

All-atom molecular dynamics (MD) simulations are a powerful tool for investigating biomolecular motion on the pico- to nanosecond time scale. While visualizing an MD trajectory provides qualitative insights into the system, quantitative data that describes or supports these insights are often difficult to obtain. In this publication we report our efforts to provide quantitative data on the essential dynamic motion of the L11·23S subdomain and the conformational metastates observed during the simulation. Specifically, we describe the use of principal component (PC) and cluster analysis.

Understanding the L11·23S dynamics and conformations will guide our efforts toward developing new active compounds that target this site. The work herein is part of a larger study concerning the binding of an antibiotic to the L11·23 subdomain [16]. However, as a first step we needed to understand how PC analysis and clustering can be used *together* for MD trajectory analysis, and what the limitations of such a combined approach might be. Thus, we explored the effects of two popular clustering algorithms on varying PC subspaces, and on the complete data as a reference, plus discuss their influence on cluster quality. An additional goal is to provide enough detail and discussion to aid others, who are new to these ideas, in their analysis of MD trajectories.

Clustering MD structural data into distinct conformational families is currently investigated by several research groups using a variety of approaches. Ideally one wants to group a molecule’s conformations according to its free energy landscape to identify kinetically metastable states. Although *kinetic clustering* [19–23] is directly capable of identifying metastable states, it requires that the transition probabilities between all microstate pairs be statistically converged [24]. Therefore, this approach is currently limited to relatively small systems or very long sampling. In contrast, *geometric clustering* is often used to analyze MD simulations since only the conformational space has to be sampled with the appropriate weights [24]. Unfortunately, geometric clustering results are dependent upon the algorithm used and may not compare well to the kinetic clustering results [24].

Alternatively, Papaoian and coworkers extracted a hierarchy of states from an energy landscape by iteratively projecting a two-dimensional histogram of the trajectory into the essential degrees of freedom identified by a PCA [25]. Approaches that combine a dimension reduction step with subsequent clustering for analyzing MD trajectory data have shown to be capable of reducing the noise and generating more compact and well separated clusters than clustering alone. Such approaches include the use of self-organizing map (SOM) [26] preclustering and quasi-anharmonic analysis extraction of metastable states [27].

Performing a PC analysis and subsequent clustering of the PC subspaces has several advantages compared to clustering the complete data set. 1) The dimensionality, and therefore time and space complexity of the clustering, is reduced considerably. On the other hand, one has to add the complexity of the PC analysis itself, which partially negates the cost saving. However, if the PCs are being calculated anyway, as in our case, the reduction in dimensionality may be worthwhile [28]. 2) Projecting the data points into PC subspace implicitly provides a native distance function for clustering: the Euclidean distance of the points in the PC subspace. 3) Plotting the resulting clusters into the most dominate PC subspaces is a useful means for visual cluster validation [29]. 4) Most importantly, PC analysis can filter out high frequency variance (i.e., “noise”) from the data and could thus, in principle, lead to a better clustering result.

Consequently, in many fields PC analysis is used as a filter prior to data clustering, allowing easier visualization of patterns within high-dimensional data [30–33]. There are a few examples where this combined analysis approach has been used to extract information from MD trajectory data [29, 34–36]; these studies found that clustering in a low dimensional PC subspace identifies approximately the same clusters as found when using full dimensions [29, 34]. However, these examples are restricted to the use of only one clustering approach (the partitional algorithms K-means [37] or the closely related K-medoid method [38]) and consider only one PC subspace. What remains to be explored is how hierarchical clustering performs, and how does clustering different numbers of PC subspaces affect the result.

## Methods

### Molecular dynamics of the ribosomal L11·23S subdomain

The 3CF5 crystal coordinates [17] were used to construct our computational model, using only the atoms associated with the ribosomal protein L11 and H43–H44 of 23S rRNA (i.e., residues 1051–1108). The resulting system was neutralized using 50 sodium ions and solvated in a truncated octahedron of 17,052 TIP3P [39] waters with a 15 Å padding in all directions. In total, the final model contained 55,186 atoms. The Parmbsc0 [40] and Parm99SB [41] force fields were used for modeling the RNA and protein.

A cutoff of 9 Å was used, while the particle mesh Ewald method [42] was employed to capture the non-bonded interactions at longer distances; 1-4 electrostatic and vdW interactions were scaled by 1.2 and 2.0. Temperature regulation was controlled using Langevin dynamics with a collision frequency of 1 ps^−1^. The SHAKE algorithm [43] was used to constrain all bonds that include hydrogen atoms. A time step of 1 fs was used during heating and increased to 2 fs during the production run; coordinates were recorded every 1 ps. A 40 ns MD trajectory of the system was obtained under constant pressure at 310 K using the AMBER software package [44]. Further details concerning the model building and simulation protocol can be found elsewhere [16].

### Principal component analysis

Principal component analysis is an unsupervised statistical technique for finding patterns in high-dimensional data. It is often used as a tool in exploratory data analysis to reveal the internal data structure in a way that best explains its variance. Initially developed about 100 years ago by Pearson [45] and Hotelling [46], it remains a popular dimension reduction technique [28].

When performed on a set of experimental structures [32, 47, 48], or snapshots from an MD trajectory [49–53], the PCs describe concerted atomic displacements and can highlight major conformational changes between the structures. Because these motions are often essential for protein function [54], the dynamics in this low-dimensional subspace – spanned by the first few PCs – was termed “essential dynamics” [49]. PC analysis, performed on Cartesian coordinates or dihedral angles (dPCA) [55, 56], has proven to be a valuable tool for studying conformational changes.

Mathematically, the PCs are obtained by a diagonilization of the data covariance (or correlation) matrix **C**.

1 $$ {\mathbf{C}} = \mathop{{{\mathbf{V}}\Lambda {\mathbf{V}}}}\nolimits^{{\mathbf{T}}} $$

This results in the diagonal matrix *Λ* containing the eigenvalues as diagonal entries and the matrix **V** containing the corresponding eigenvectors. If the eigenvectors are sorted such that their eigenvalues are in decreasing order, the eigenvector with the largest eigenvalue (i.e., the first PC) accounts for the highest proportion of variance within the data, the second component is orthogonal to the first one and accounts for the second highest proportion of variance, and so on. If there is a high signal-to-noise ratio in the data, the first few PCs can be considered, while the rest can be neglected without significant loss of information. It is also important to note that PC analysis assumes linearity, which means it is limited to reexpressing the data as a linear combination of its basis vectors.

### Clustering

Cluster analysis is another unsupervised technique for finding patterns within data. Clustering algorithms group similar objects into subgroups (i.e., clusters) by minimizing intra-cluster and maximizing inter-cluster differences. Therefore, most clustering algorithms require a measure of similarity, or “distance”, of objects. Clustering algorithms can be divided into *partitional* and *hierarchical* clustering approaches [57]. Partitional clustering is a division of the objects from the data set, for example different conformations from an MD trajectory, into non-overlapping clusters; hierarchical clustering allows nested clusters and results in a hierarchical tree (i.e., dendrogram). They are either bottom-up *agglomerative* approach or top-down *divisive* approach. A partitional clustering can then be obtained by cutting the dendrogram at a particular level. The average value within a cluster is called the centroid; for clustering coordinate data, the centroid represents the conformation that best describes the conformations within a cluster.

In the following subsections we will provide a brief background concerning partitional and agglomerative hierarchical clustering approaches. Ultimately, we chose K-means and average-linkage as representative partitional and hierarchical clustering algorithms due to their apparent good performance in analyzing MD trajectory data and their frequent availability in MD analysis packages [58].

### Partitional clustering

Partitional techniques optimize a locally or globally defined criterion function to determine a *pre-specified* number of clusters, most often using the squared error criterion [59]. Of these, the K-means algorithm is one of the oldest, fastest, and most widely used techniques. The algorithm places a predefined number of initial centroids (*K*) randomly. In an iterative process, *K* clusters are formed by assigning each data point to its closest centroid and a new centroid for each cluster is recomputed. These steps are repeated until the cluster membership is stable (i.e., converged). However, the result depends on the initially chosen centroids (i.e., not deterministic). This method can fail when the “natural” clusters have non-spherical shapes, widely different sizes or densities, or when the data contains outliers [57]. Performed on MD trajectory data, K-means tends to produce “blocky” clusters of similar sizes [58].

### Agglomerative hierarchical clustering

Agglomerative hierarchical clustering is a collection of closely related techniques that start with singleton clusters and proceeds to iteratively join the nearest cluster until all objects are grouped into one cluster [57]. The various styles within this technique differ in their definition of cluster proximity. *Single-linkage* defines cluster proximity as the “closeness” between the nearest two objects that are in different clusters, *complete-linkage* uses the farthest two objects, and *average-linkage* uses the average pairwise proximities of all pairs of objects from different clusters. *Centroid* methods use the distance between the centroids of clusters to define cluster proximity. Finally, in *Ward*’s method the proximity is defined as the increase in the squared error that results when two clusters are merged [60]. Of these styles, the centroid and average-linkage are found to be the most useful approaches for analyzing MD trajectory data [58].

Agglomerative hierarchical clustering algorithms are deterministic, allowing for reproducibility of resulting clusters. These algorithms produce a dendrogram displaying the cluster-subcluster relationships and the order in which the clusters were merged. Unfortunately, their high computational and storage requirements limit their use to smaller data sets.

### Metrics for cluster validation

Unsupervised methods are difficult to assess as they do not provide a native evaluation criterion. Although important, cluster evaluation is not commonly used in cluster analysis [57]. There exist several different metrics that give some general quantitative indication on cluster quality [57, 61, 62]. Each of them has their specific drawbacks and there is no consensus on which method should preferentially be used. In this study we used two metrics that have been shown useful before [58].

A simple measure to determine the optimal number of clusters is to calculate the *SSR/SST ratio*, the quotient of the sum of squares regression (SSR or between sum of squares) and the total sum of squares (SST). The SSR is usually calculated via the sum of squares error (SSE or within sum of squares) – that is the sum of the squared distances of all points within cluster *C*_*i*_ to its centroid *c*_*i*_, and summed together for all clusters *K*. The total sum of squares is the sum of squared distances for all data points to the overall mean *c* and is equivalent to the SSE if *K* is 1.

2 $$ SSE = SST - SSR = \sum\limits_{{i = 1}}^K {\sum\limits_{{x \in {C_i}}} {{{\left( {x - {c_i}} \right)}^2}} } $$

3 $$ SST = \sum\limits_{{j = 1}}^N \,{\left( {{x_j} - c} \right)^2} $$

The SSR/SST ratio value lies between 0 and 1 and gives the percentage of explained variance by the data, and is similar to the *R* ^2^ value in regression analysis. As the ratio inherently rises with cluster count, one looks for an “elbow” in the curve where adding another cluster does not add much new information, as done in a scree test.

As a second metric we calculated the *pseudo F-statistic* (Eq. 4, introduced by Caliński and Harabasz [63]). This metric is a measure for the “tightness” of clusters; high values usually indicate a better clustering.

4 $$ pFS = \frac{{SSR/K - 1}}{{SSE/\left( {N - K} \right)}} $$

PC analysis, clustering, and clustering metrics were done with R using the bio3D package [64], which was specifically developed for analyzing biomolecular data. Only the atomic coordinates of the C*α* and phosphorus atoms (199 total atoms) were used in the analysis to reduce statistical noise and to avoid artificial apparent correlations between slow side-chain fluctuations and backbone motions. A PCA in dihedral angle space is based on internal coordinates which naturally provide a correct separation of internal and overall motion. However, dPCA can miss relevant motions since major collective dihedral transitions do not usually correspond to major transitions in Cartesian space [54]. Moreover, visualization of the resulting principal components – often essential for their meaningful interpretation – is problematic as the configuration space cannot be retained in a straightforward way. Therefore, we decided to do our PC analysis based on Cartesian coordinates. The trajectory snapshots were aligned to the X-ray structure using these atoms in order to remove trivial rotational and translational movements prior to the analysis. Due to the high storage demand of the average-linkage algorithm, not all snapshots recorded during the MD simulation could be included into the clustering. Therefore, a reduced data set, containing only every fifth snapshot, was clustered with both algorithms. A test with K-means on the complete snapshot set showed that this did not influence the clustering outcome (data not shown).

## Results and discussion

### Principal component analysis

A substantial equilibration time was required for the model due to the L11·23S subdomain’s extraction from the complete ribosomal crystal structure, its immersion into water, and the removal of a bound ligand (i.e., thiostrepton). Equilibration was established by monitoring the system’s RMSD and radius of gyration [16] (Supplementary Fig. S1 and S2). Consequently, the time frame from 2.5 to 40 ns was considered as production data in order to fully remove the model’s equilibration period. The resulting 597 × 37,500 data matrix was subjected to a principal component analysis, yielding 3 × 199 principal components (i.e., eigenvectors). The six eigenvectors that correspond to the smallest eigenvalues describe the overall translation and rotation of the system, were essentially zero after snapshot alignment.

**Fig. 1:**
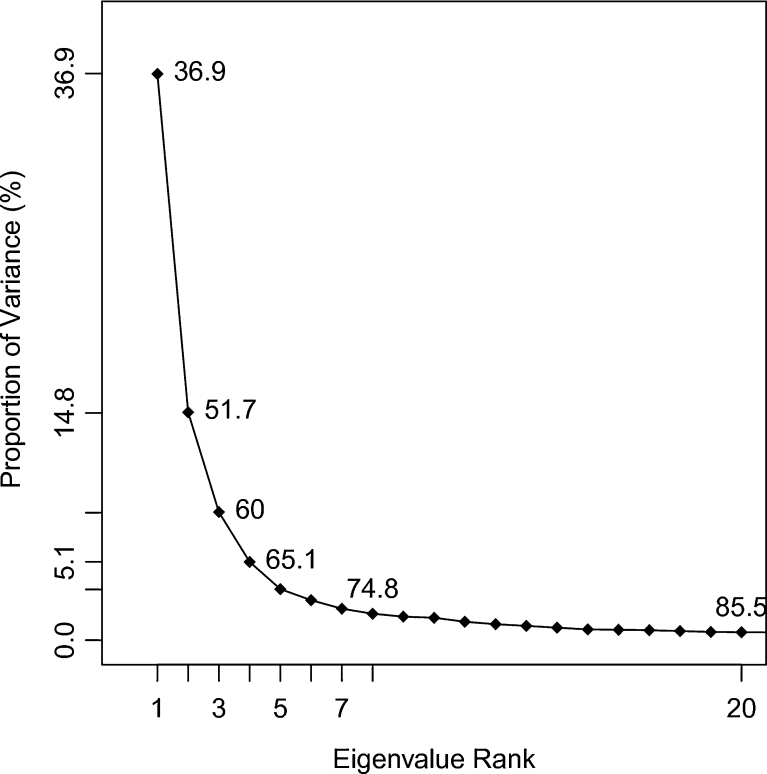
Scree plot for principal component analysis on the MD coordinate data of the L11·23S ribosomal subdomain

Figure 1 shows the resulting PC analysis scree plot, indicating the proportion of variance against its eigenvalue rank. The first PC accounts for more than one third of the overall variance, strongly dominating the overall variance. The first three components together make up 60 % of the variance. Afterward, the individual component contributions drops below 5 %.

Using the bio3D function *mktrj.pca* [64], interpolated trajectories of atomic displacements along the first five PCs were visually inspected. The first PC describes a twisting motion of L11’s N-terminal domain with respect to the RNA. The second PC is dominated by loop motion within the N-terminal domain. The remaining components did not allow for a clear visual interpretation (see Supplementary Animation 1 for the first three PCs).

**Fig. 2:**
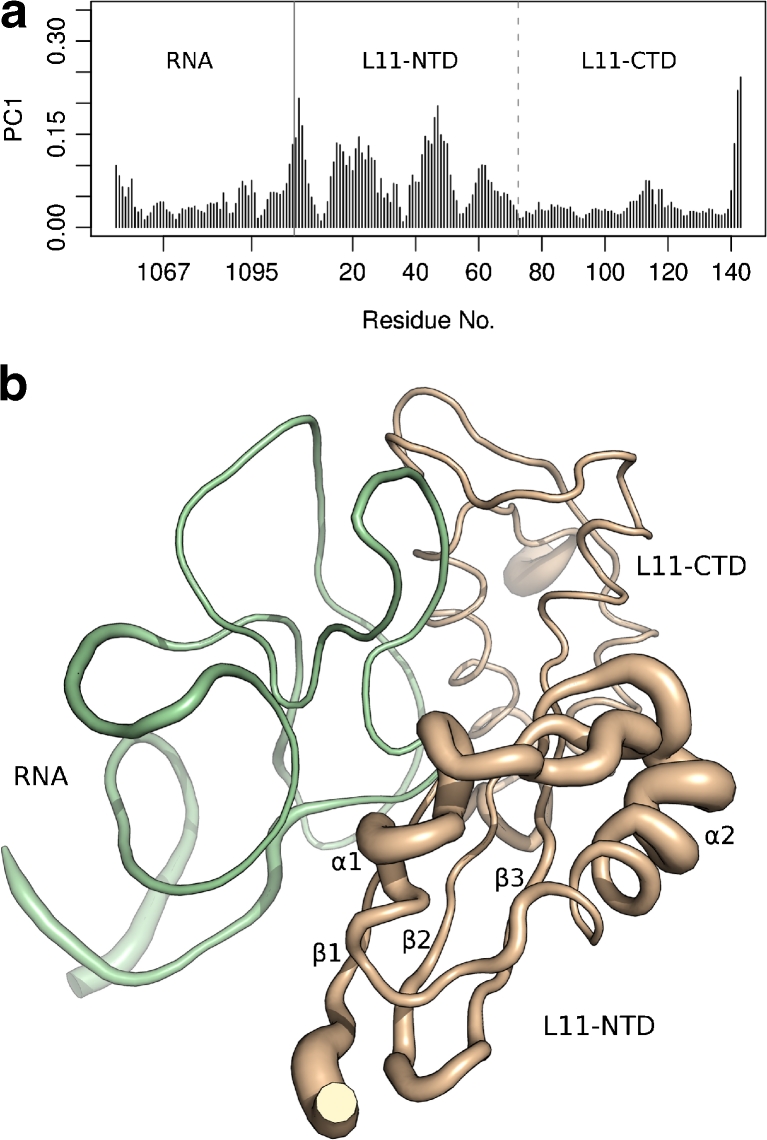
L11·23S subdomain’s residue contributions to the first PC. **a** Residue-wise loadings (i.e., contributions) in Å to the first PC. The black solid line separates RNA and protein residues, the dashed line separates the N- and C-terminal domain of L11. **b** Residue contributions to the first PC is shown by thickness as mapped onto the 3CF5 coordinates. The RNA is colored green, the L11 protein is colored wheat. Figure created using PyMOL [69]

Another technique for guiding the interpretation of PC analysis results is to attribute the individual residue contributions to the PCs. Figure 2 shows these contributions for the first PC in a (a) graphical and (b) structural depiction. It can be seen that the RNA and C-terminal protein domains contribute little, except for the chain endings. The contributions from the protein termini may be real since they do not participate in a stable secondary structure [16]. Conversely, the contribution of the RNA terminal residues are likely artifacts arising from the creation of our truncated model. The highest loadings are observed in the distal end of the N-terminal protein domain, and in the loop proceeding *α*2 (see Fig. 2(b)).

**Fig. 3:**
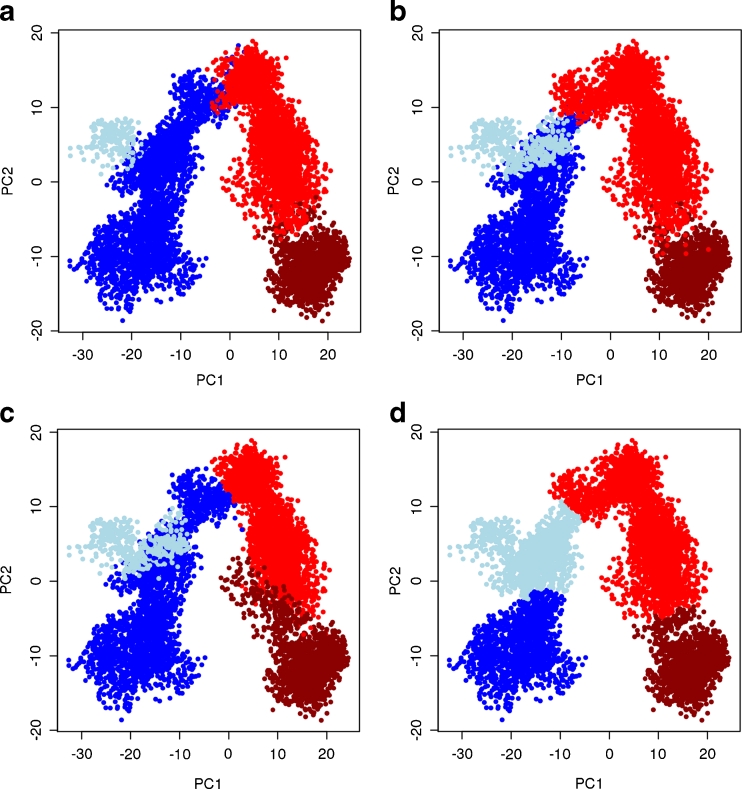
Clustering results of average-linkage algorithm on different subspace dimensions projected on 2d plane formed by first two PCs. Clustering was performed on the entire MD trajectory data (**a**), and on the data from the first five (**b**), three (**c**), and two (**d**) principal components. Key: cluster 1 is blue, cluster 2 is lightblue, cluster 3 is red, and cluster 4 is darkred

**Fig. 4:**
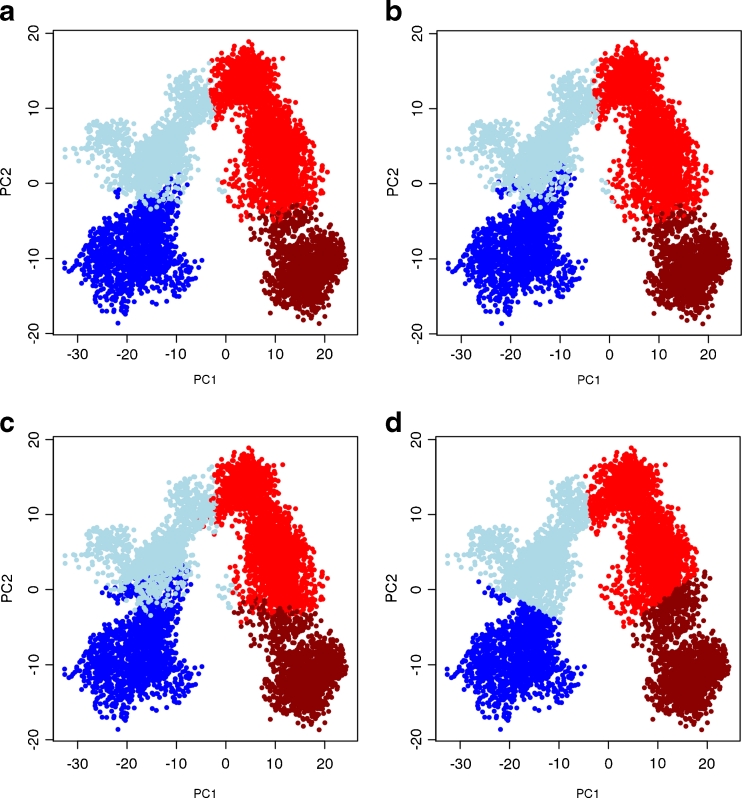
Clustering results of K-means algorithm on different subspace dimensions projected on the 2d plane formed by the first two PCs. Four clusters were requested in the computation. Clustering was performed on the entire MD trajectory data (**a**), and on the data from the first five (**b**), three (**c**), and two (**d**) principal components. The colors correspond to the cluster as defined in Fig. 3

Projecting the trajectory snapshots onto the plane formed by the first two principle components reveals a semicircle, or U-shape, relationship (Figs. 3 and 4). As discussed by Hess, such a pattern probably indicates random diffusion within the simulation, and is interpretable as thermal motion along a shallow free-energy landscape [65, 66]. While such a result does not inform us of dominating large-scale conformational changes within the system, it does inform us of the more accessible degrees-of-freedom for thermal motion along our investigated time scale [65].

While PC analysis reveals the main motions contained in an MD trajectory it does not partition the snapshots into distinct conformational classes. This can be achieved by clustering the PC data. In the following, we will investigate if clustering PC analysis data improves cluster quality versus clustering the “raw” data set, and how two different clustering algorithms perform. The resulting clusters allows for comparisons to be made between conformations that are sampled during the apparent thermal diffusion.

### Influence of subspace

Clustering can identify different conformational states sampled during the simulation by grouping molecular structures into subsets based on their conformational similarity [67]. This requires a definition of (dis)similarity by a distance metric and a definition of the space where the clustering should occur. In this study, we will restrict ourselves to the Euclidean distance expressed by the root-mean-square deviation (RMSD) between conformations, and focus on the different options for the subspace that will be used for clustering. The most straightforward, which is also the most computationally expensive, is to cluster the complete trajectory data set. Since we are initially interested in the large-scale motion of the L11·23S subdomain, we chose to exclude the side-chain local motion; this was achieved by using only the coordinates of the protein’s C*α* and RNA’s phosphorus backbone atoms. By considering these atoms only, a reduction in the raw data analyzed is also obtained, making computation more tractable.

Clustering can also be performed on an even further reduced subset, such as the data contained within the first few PCs, where the high-frequency motion and “noise” has been removed. The assumption present in this combined technique is that the clustering focuses on data that is more relevant to large-scale motion within the interested molecular species. Consequently the question arises, “How many components should be included into the cluster analysis?” Common subsets of PCs, typically analyzed in PC analysis, are the first two or three PCs since most of the overall variance is often captured within the first two or three components (e.g., 51.7 % and 60 %, respectively, as seen in Fig. 1). Moreover, these subspaces can still be visually interpreted. We also decided to include the fifth-dimensional subspace based on the scree test [68] – the fifth PC is where the percentage of variance becomes relatively horizontal as shown in Fig. 1, a criterion known as the “elbow-criterion.” In summary, clustering was performed on two-dimensional (2d: PC one and two), three-dimensional (3d: PC one to three), five-dimensional (5d: PC one to five) subspaces, and on the complete data set (Figs. 3 and 4).

**Fig. 5:**
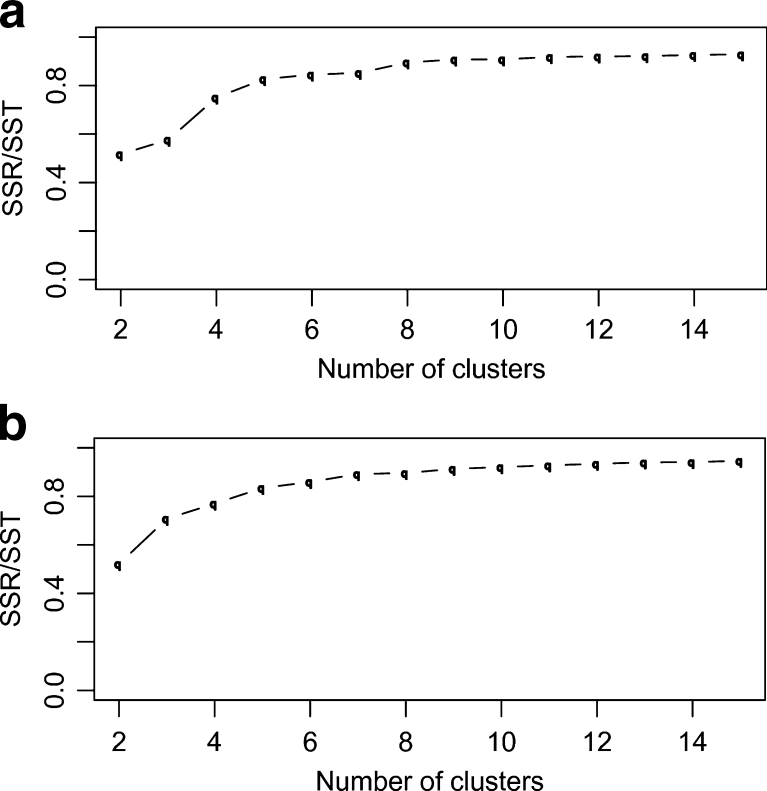
SSR/SST ratio over cluster count for the entire MD trajectory data. **a** average-linkage, **b** K-means algorithm

Another important question is, “How many significant clusters are there?” Determining the number of clusters in the data is a frequent problem in cluster analysis, especially for algorithms such as K-means that requires this value as an input parameter. We calculated the SSR/SST ratio as a statistical measure to determine the optimal number of clusters for both algorithms whose values are plotted in Fig. 5. Both algorithms show an SSR/SST ratio of ~0.5 at a cluster count of two which means that the data’s variance is already halved by choosing two subsets. Thus, the data clearly supports at least two clusters. Another increase is found at a cluster count of four although a clear “kink” cannot be identified, leaving a choice of four somewhat arbitrary. However, the same conclusions can be drawn from a visual inspection of the average-linkage’s cluster dendrogram (Supplementary Fig. S3). Overall, both approaches indicate a cluster count of four is reasonable.

**Fig. 6:**
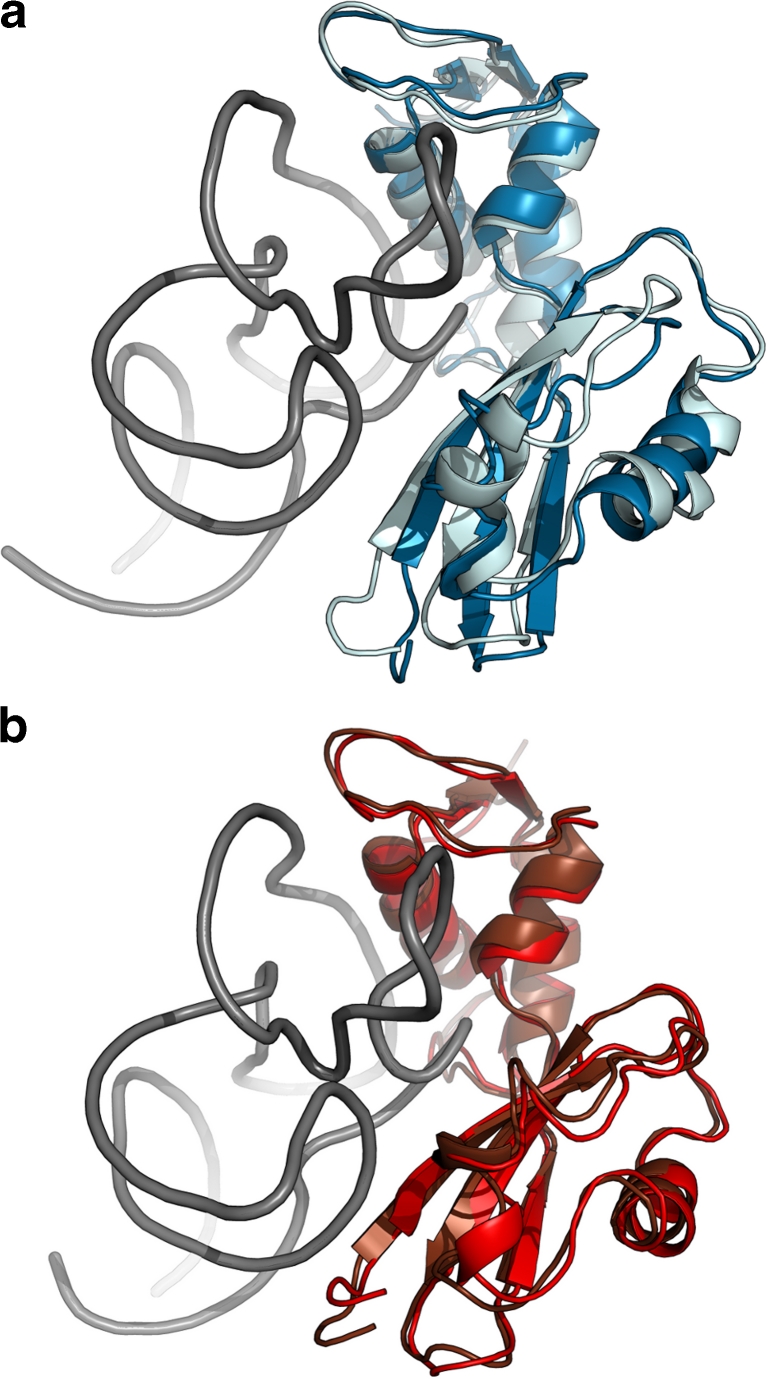
Overlay of the best representative structure (i.e., structure with lowest RMSD in comparison to the cluster’s centroid) for the four cluster found using K-means clustering of the 3d PC subspace. **a** Clusters 1 and 2, **b** clusters 3 and 4. For clarity, the RNA (gray), displaying minor structural differences, is shown for cluster 1 (**a**) and 3 (**b**) only

In addition to analyzing the cluster dendrogram we considered the separation of data points along the axes of the first three PCs (see Supplementary Animation 2) as a second visual criterion. This visualization confirmed the choice of four clusters, showing that cluster 1 (blue) and 3 (red) are clearly separated by the first PC. The second PC further separates cluster 2 (lightblue) and 3 from 1 and 4 (darkred). Thus, from the SSR/SST ratio and the visual analysis we concluded that the trajectory contains four conformational classes – two major conformations and two minor conformations, which can be seen in Fig. 6.

**Fig. 7:**
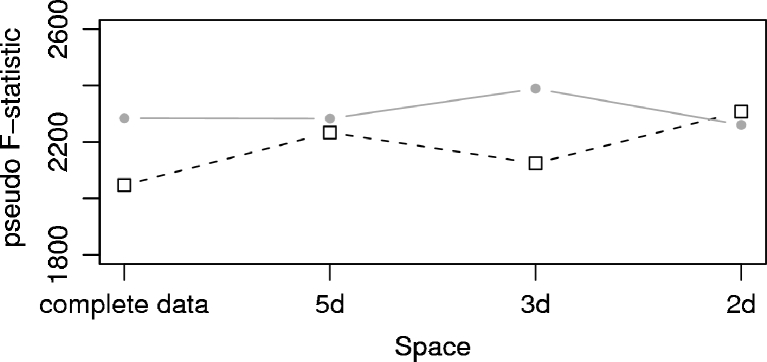
Pseudo F-statistic for clustering in different subspaces. A fixed number of four clusters was used. Black squares: average-linkage, gray circles: K-means algorithm

In a similar fashion, by combining an objective statistical measure with different visual analytics, we evaluated the clustering in the different PC subspaces. Figure 7 shows the pseudo F-statistic for the two clustering algorithms. To compare the absolute *pFS* values, we kept the number of clusters fixed (i.e., four) and calculated the *pFS* on the complete data set, taking the clusters as obtained from clustering in the different subspaces. For K-means the values are very similar – the *pFS* value of the 2d and 5d subspace and of the complete data are essentially the same. Only for the 3-dimensional subspace a small rise in the *pFS* value (i.e., a better clustering) can be seen. For clustering using the average-linkage algorithm, the *pFS* values are lower than for K-means with the exception seen in the 2d case. Moreover, the clustering quality across the different PC subspaces shows higher variation when average-linkage is used. Note that these statistical observations may be different when investigating other molecular systems and should be evaluated accordingly.

Substantial differences in data-point assignments to different clusters are seen in using the average-linkage algorithm when performed on different dimensions of PC subspaces (Fig. 3). Especially the size of cluster 2 (lightblue) changes. Cluster 2 is very small in favor of a large cluster 1 (blue) when clustering the complete data (a), is medium-sized for 5d (b) and 3d (c) which differ in their boundary assignments between cluster 1 (blue) and 3 (red), and is the largest in the 2d case (d). In contrast, the K-means results are considerably more consistent, regardless if clustering was done using the complete data set or any number of PCs studied (Fig. 4 (a–d)). Only a few data points are affected, corresponding to data within the K-means cluster boundary regions.

Thus, the data-point membership to a specific cluster depends more on the selected subspace when using average-linkage clustering than for K-means. K-means appears to be more robust in this regard. Interestingly, selecting a subspace defined by the first two PCs (Figs. 3(c) and 4(c)) yield comparable clustering results for both algorithms and is the only condition that leads to a better clustering result (via pseudo F-statistics) for average-linkage. Whether or not this is a fortuitous agreement is currently unclear.

**Fig. 8:**
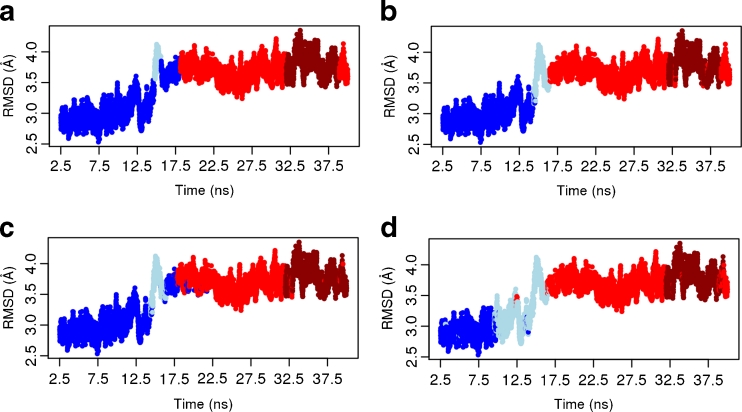
RMSD over simulation time and color-coded by clusters obtained from average-linkage algorithm for the the complete data (**a**), the first five PCs (**b**), the first three PCs (**c**) and the first two PCs (**d**). The colors correspond to the cluster as defined in Fig. 3

As an additional way to verify and interpret the clustering results, we graphed the root-mean-square deviation (RMSD) versus simulation time, and color-coded the data points by cluster membership as determined using the average-linkage (Fig. 8) and K-means (see Supplementary Fig. S4) algorithms on the different PC subspaces. In contrast to Figs. 3 and 4, these plots visualize the clustered snapshots over time along with their deviation from a defined reference structure, which is the 3CF5 X-ray structure. If a dynamic event occurs during the simulation, which changes the structure’s conformation, then the clustering algorithms should be able to distinguish these conformations.

As expected from Figs. 4 and 7, hardly any differences in snapshot assignment to a K-means cluster are observable for the different subspaces (also see Supplementary Fig. S4). For average-linkage, however, the distribution of clusters over simulation is divisive (Fig. 8). Clearly separated in all four cases are cluster 1 (blue) at the beginning of the simulation (after equilibration), cluster 3 (red) in the middle, and cluster 4 (darkred) at its end. Disagreement occurs in their lifetime and in the conformational snapshots assigned to cluster 2 (lightblue). Depending on the data clustered, cluster 1 membership can be fragmented across a dynamic event (Fig. 8(a) and (c)). Thus, average-linkage clustering of different PC dimensions require careful analysis since the snapshots cannot be unambiguously assigned. In this study, the clustering in the subspace defined by the first two PCs (Fig. 8(d)) provides the most coherent picture. Moreover, this clustering is supported by a good *pFS* value and is almost identical to each K-means result (Supplementary Fig. S4).

Returning to our initial statement of providing quantitative data to support the qualitative observations, we visually observed that the protein’s NTD was dynamically mobile during the simulation, while the CTD and rRNA were fairly inflexible. Performing a PC analysis and clustering the resulting data revealed four distinct clusters of the L11·23S subdomain – two major and two minor clusters. From the RMSD analysis, the structures of the first cluster have conformations that are similar to the crystal structure. The second cluster is best described as a conformational transition from cluster one to another stable conformation sampled by cluster three. Cluster three remains stable for ~15 ns before a closely related conformation (cluster 4) is sampled, which lasts for the remainder of the simulation. Figure 6 displays the structural representatives of the four clusters, whose differences can be described as a rotational movement of the NTD with respect to the CTD and rRNA. Their main differences are in the position of the N-terminus, the nearby loop connecting the *β*2-*β*3 strands, and the two loops connecting the helices with the *β*-strands on the far end (i.e., away from the binding site) of the N-terminal domain. As mentioned earlier, these conformations are representative structures that lie along the thermal relaxation pathway as sampled during our simulated time frame.

## Conclusions

Researchers who perform MD simulations and wish to “organize and conceptualize” [24] the resulting conformational data must address how to best sift this information. Generally speaking there exists two approaches for sifting conformations into meaningful organized groups, which are broadly referred to as kinetic clustering [19–23] and geometric clustering [24]. The details of how the clustering is performed and how clustering can be combined with other simulation observables (e.g., free energy, PCA) remains an active area of research.

In this study we used PC analysis combined with clustering to study a portion of the ribosomal L11·23S subdomain dynamics. A PC analysis on an MD trajectory data revealed that the major motions during the simulation are dominated by the N-terminal domain, whose dynamic behavior is believed to be important for protein translation [3, 6, 13, 17]. By a subsequent clustering using the K-means algorithm of the structural snapshots in the subspace spanned by the first three PCs we identified distinct conformational classes mainly differing by the orientation of the N-terminal domain.

We compared two widely used clustering methods, each representing a different clustering approach: K-means (partitional) and average-linkage (hierarchical). In our study, K-means slightly outperformed average-linkage. K-means results gave better clustering statistics and provided more consistent clustering results (i.e., data-point membership to specific clusters, cluster shape and size) using the different dimensions of PC subspace. Drawbacks to its use include its need for a predetermined clustercount and its tendency to form blocky, spherical clusters [57]. If the underlying data do not support this cluster structure, K-means will not provide good results.

The clusters found by average-linkage can be of varying size and shape, and the cluster dendrogram that hierarchical algorithms naturally provide proved valuable for determining the optimal cluster count. We found that the outcome of the average-linkage algorithm was strongly dependent on the selected PC subspace. In our L11·23S model, the choice of the 3d subspace defined by the first three PCs combined with the K-means algorithm provided the best clustering results, even in comparison to using the original data.

In conclusion, we found that using both clustering algorithms to analyze different PC subspaces allowed us to form a coherent conclusion concerning the number of clusters present in our MD trajectory data. Mapping these clusters onto 2d and 3d plots of the first two and three PCs, and onto an RMSD versus time plot, allowed us to understand the clusters’ time and conformational space relationship better. Due to our examination of a single model system, generalization concerning the clustering of different PC subspaces of other molecular systems would be premature at this time.

## Electronic supplementary material

Below is the link to the electronic supplementary material.

Animation 1 A movie of the interpolated trajectories of the first three principal components, as provided by bio3D’s *mktrj.pca* (see http://thegrantlab.org/bio3d/index.html) algorithm. (MPG 811 kb)

Animation 2 A three-dimensional plot of every tenth data point along the axes of the first three PCs and clustered using the K-means algorithm. Key: cluster 1 is blue, cluster 2 is light blue, cluster 3 is red, and cluster 4 is dark red. (MPG 1636 kb)

Figure S1 RMSD over the entire simulation, showing the initial 2.5ns equilibration time period. The orange line represents the smoothing of individual data points (black dots) using Bezier curves. (PNG 65 kb)

Figure S2 Radius of gyration over simulation time. The orange line represents the smoothing of individual data points (black dots) using Bezier curves. (PNG 86 kb)

Figure S3 Clustering dendrogram produced by the agglomerative average-linkage algorithm using the entire MD trajectory data. The blue rectangles denote the cut to obtain two different clusters, the red rectangles denote four different clusters. (PNG 76 kb)

Figure S4 RMSD over simulation time and color-coded by clusters obtained from K-means algorithm for the different PC subspaces and the complete data. Key: cluster 1 is blue, cluster 2 is light blue, cluster 3 is red, and cluster 4 is dark red. (PNG 50 kb) (PNG 52 kb) (PNG 52 kb) (PNG 53 kb)
