# Supplementary figures and images for: Principal component and clustering analysis on molecular dynamics data of the ribosomal L11·23S subdomain
Source: J Mol Model. 2012 Sep 8;19(2):539–49. doi: 10.1007/s00894-012-1563-4 (PMC3592554; doi:10.1007/s00894-012-1563-4)

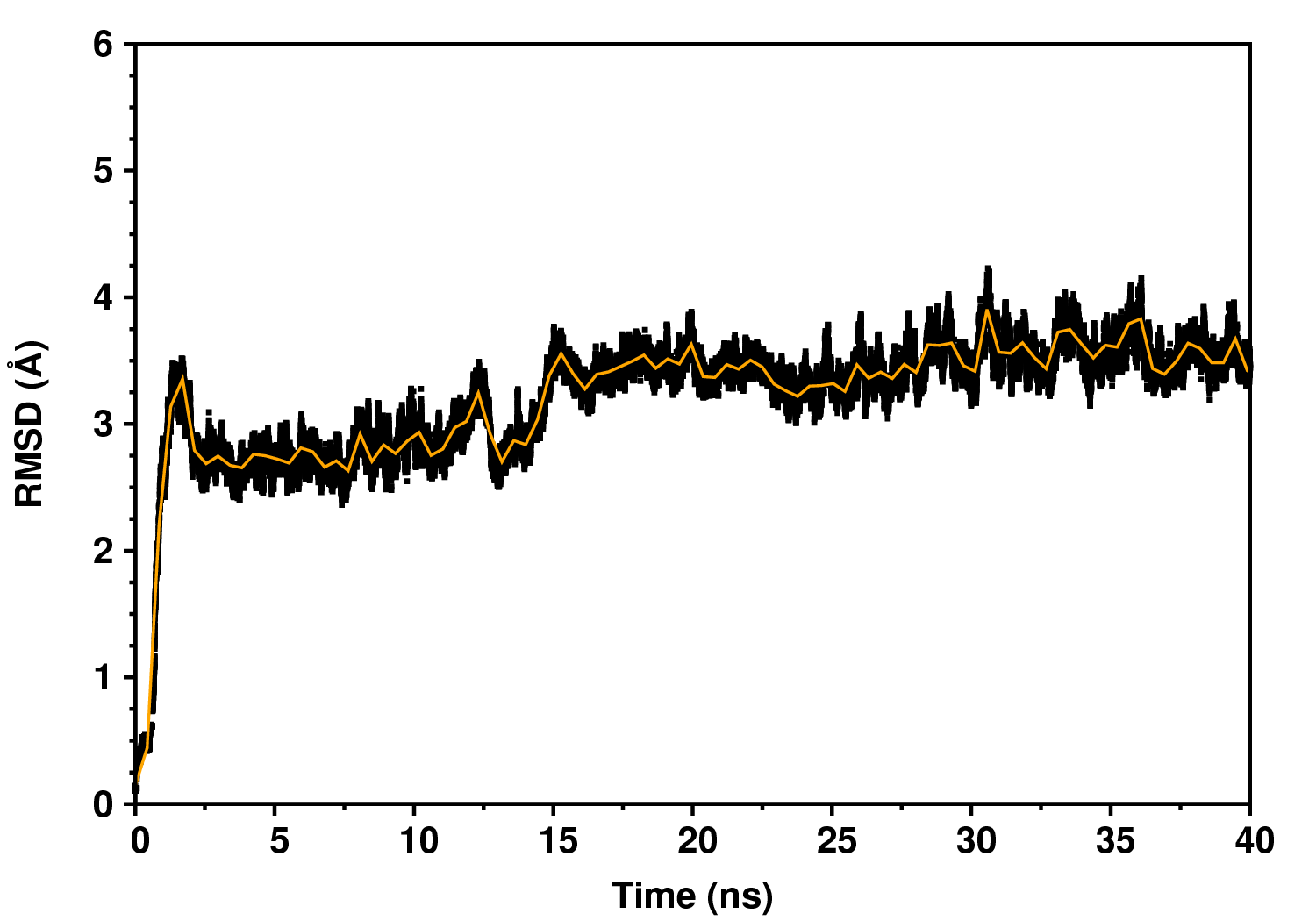

Supplement: Supplementary file 3 — RMSD over the entire simulation, showing the initial 2.5ns equilibration time period. The orange line represents the smoothing of individual data points (black dots) using Bezier curves. (PNG 65 kb) [file 894_2012_1563_MOESM3_ESM.png]

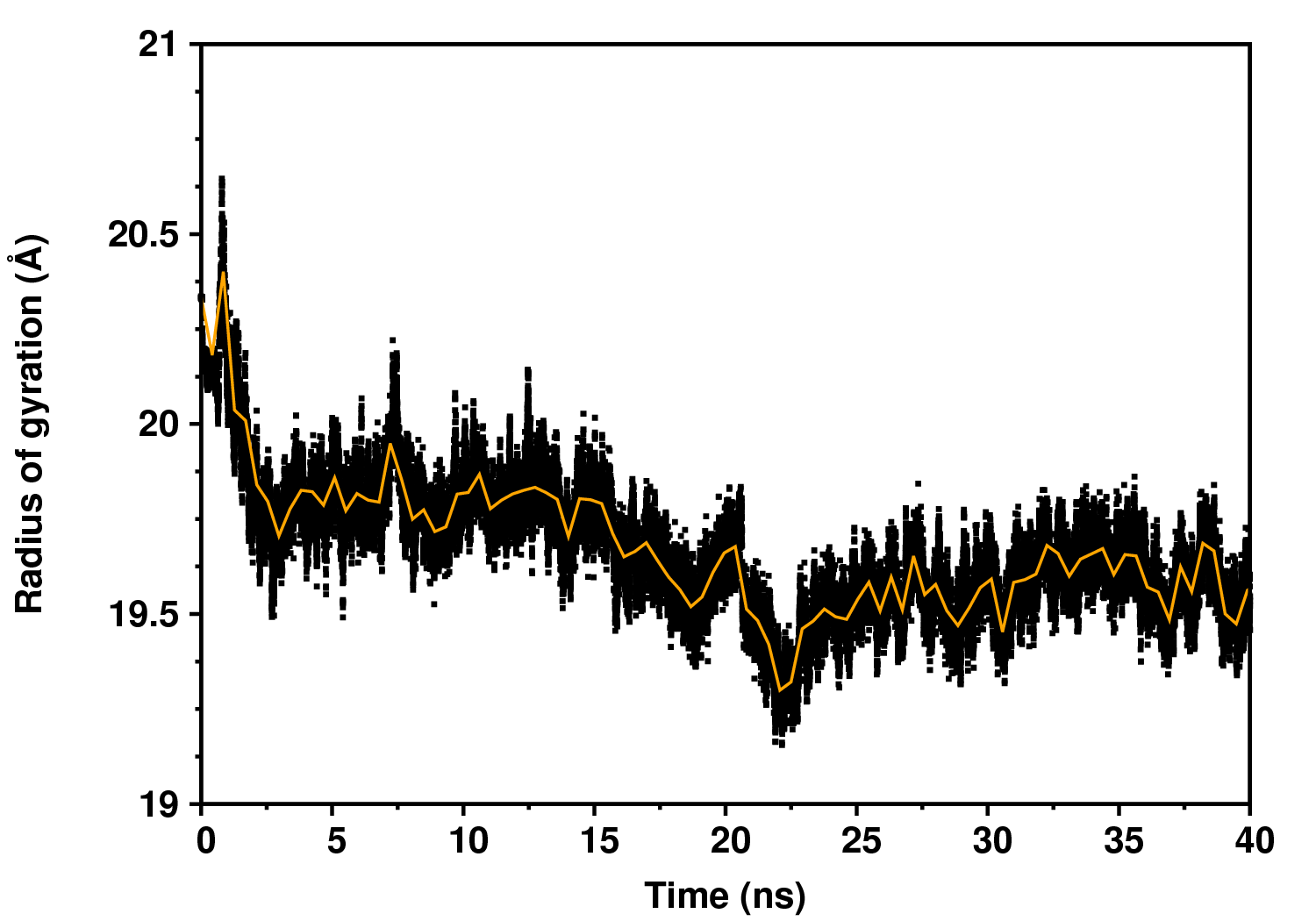

Supplement: Supplementary file 4 — Radius of gyration over simulation time. The orange line represents the smoothing of individual data points (black dots) using Bezier curves. (PNG 86 kb) [file 894_2012_1563_MOESM4_ESM.png]

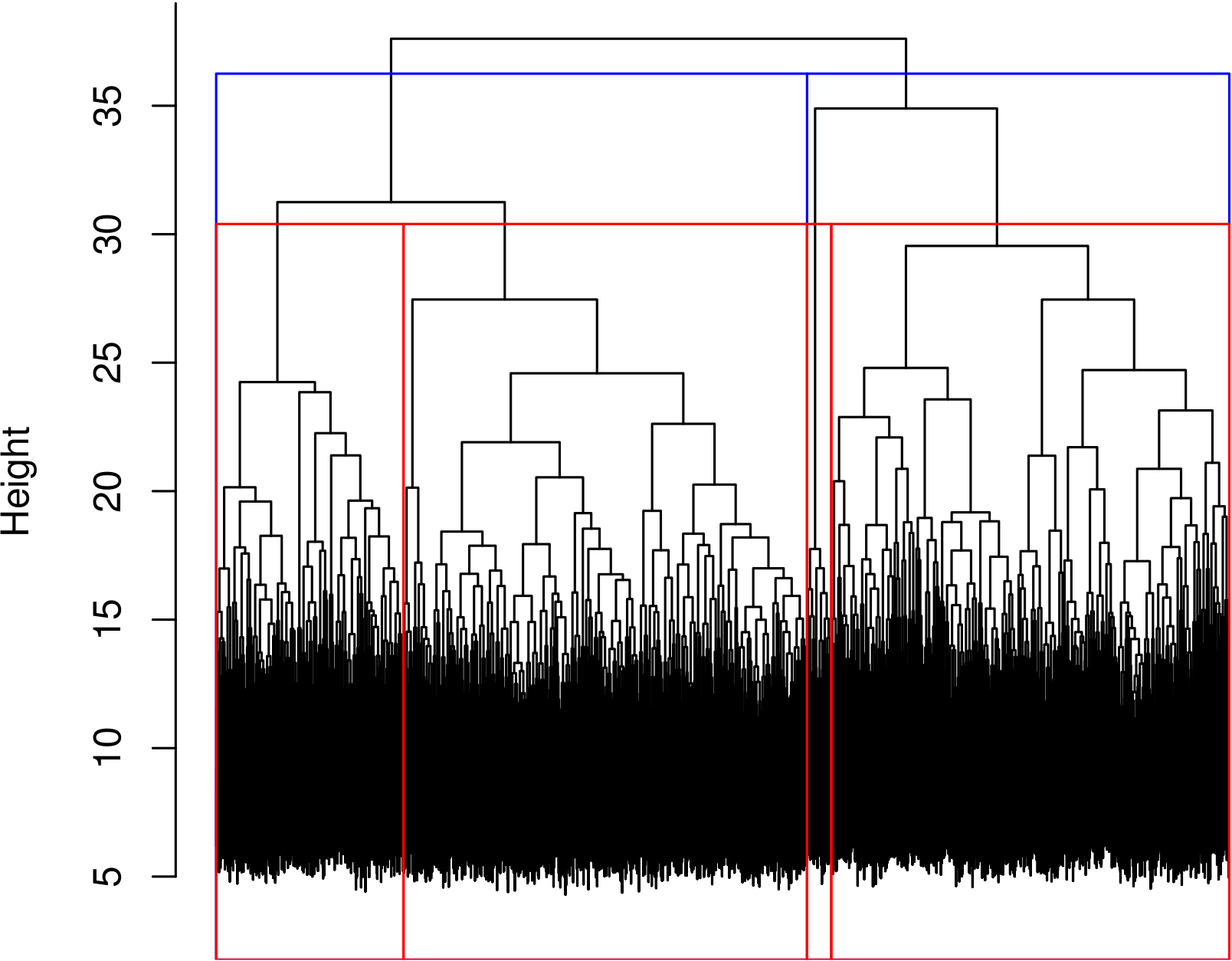

Supplement: Supplementary file 5 — Clustering dendrogram produced by the agglomerative average-linkage algorithm using the entire MD trajectory data. The blue rectangles denote the cut to obtain two different clusters, the red rectangles denote four different clusters. (PNG 76 kb) [file 894_2012_1563_MOESM5_ESM.png]

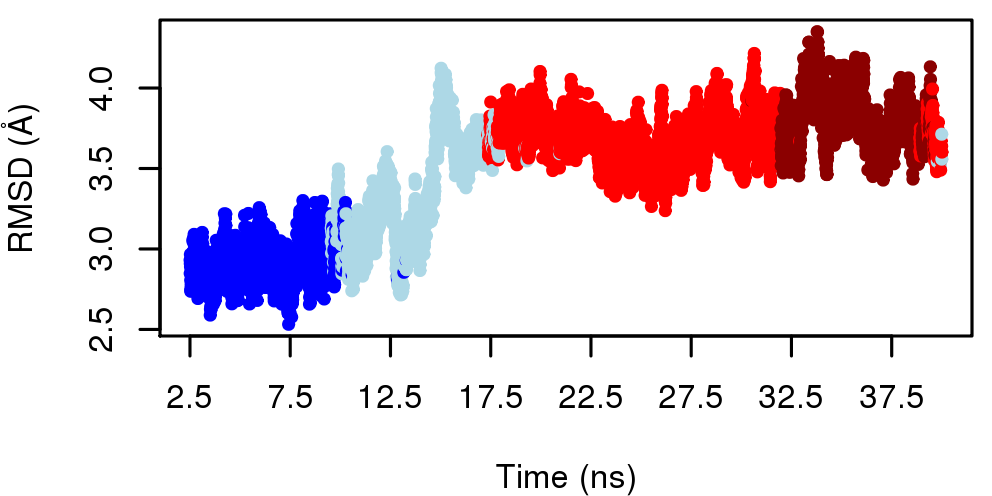

Supplement: Supplementary file 6 — RMSD over simulation time and color-coded by clusters obtained from K-means algorithm for the different PC subspaces and the complete data. Key: cluster 1 is blue, cluster 2 is light blue, cluster 3 is red, and cluster 4 is dark red. (PNG 50 kb) [file 894_2012_1563_MOESM6_ESM.png]

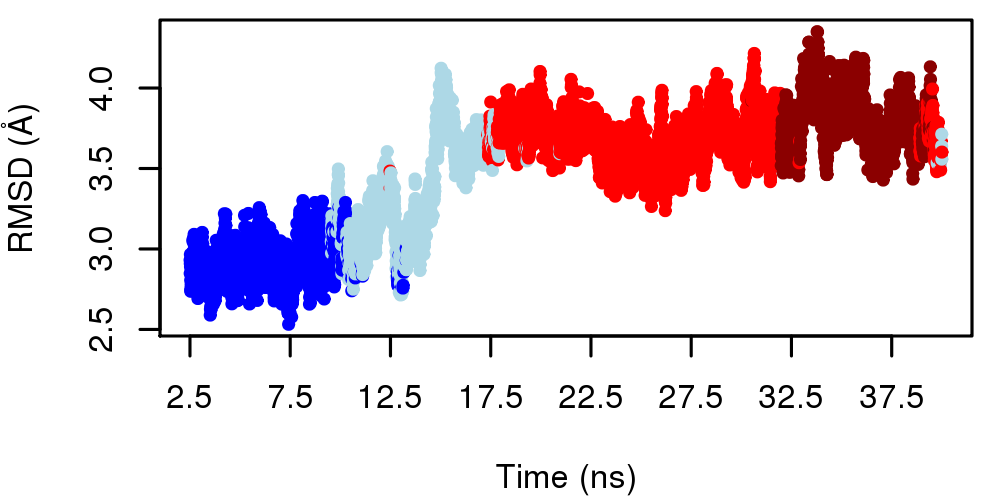

Supplement: Supplementary file 7 — (PNG 52 kb) [file 894_2012_1563_MOESM7_ESM.png]

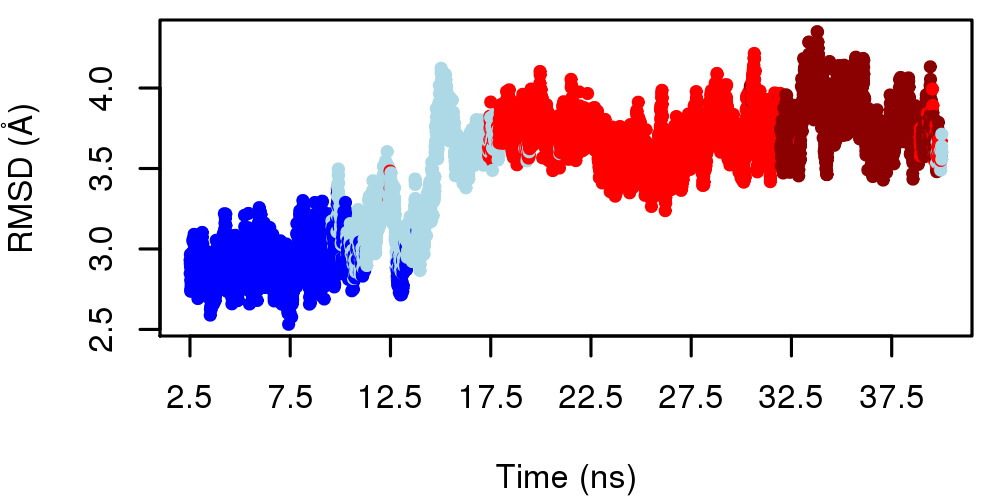

Supplement: Supplementary file 8 — (PNG 52 kb) [file 894_2012_1563_MOESM8_ESM.png]

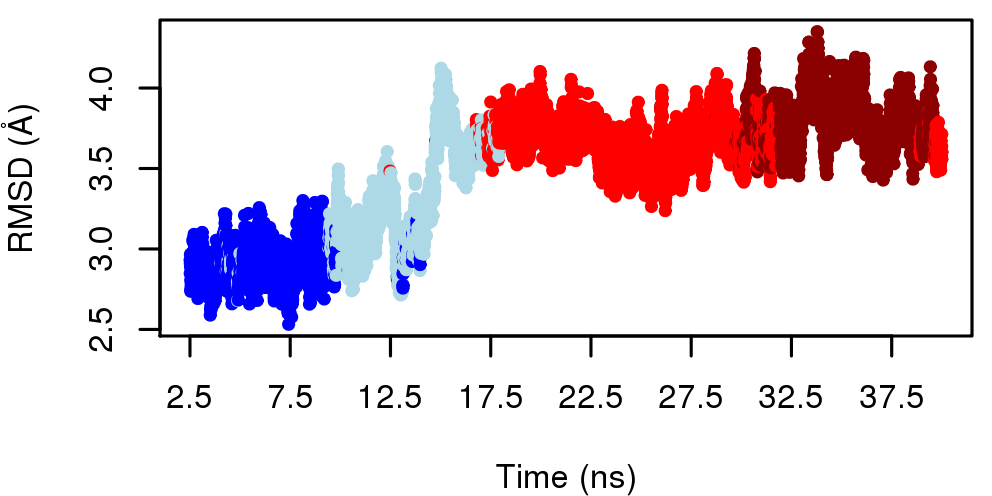

Supplement: Supplementary file 9 — (PNG 53 kb) [file 894_2012_1563_MOESM9_ESM.png]
